# PCR-based screening, isolation, and partial characterization of motile lactobacilli from various animal feces

**DOI:** 10.1186/s12866-020-01830-7

**Published:** 2020-06-03

**Authors:** Shunya Suzuki, Koki Fujita, Shintaro Maeno, Yuh Shiwa, Akihito Endo, Kenji Yokota, Shizunobu Igimi, Akinobu Kajikawa

**Affiliations:** 1 Department of Agricultural Chemistry, Graduate School of Agriculture, Tokyo University of Agriculture, 1-1-1 Sakuragaoka, Setagaya, Tokyo, 156-8502 Japan; 2 Department of Food, Aroma and Cosmetic Chemistry, Graduate School of Bioindustry, Tokyo University of Agriculture, 196 Yasaka, Abashiri, Hokkaido 099-2493 Japan; 3 Department of Molecular Microbiology, Tokyo University of Agriculture, 1-1-1 Sakuragaoka, Setagaya, Tokyo, 156-8502 Japan; 4 Department of Food, Aroma and Cosmetic Chemistry, Tokyo University of Agriculture, 196 Yasaka, Abashiri, Hokkaido 099-2493 Japan; 5 Department of Agricultural Chemistry, Tokyo University of Agriculture, 1-1-1 Sakuragaoka, Setagaya, Tokyo, 156-8502 Japan

**Keywords:** Motile lactobacilli, *Lactobacillus agilis*, Isolation, PCR detection, Animal feces

## Abstract

**Background:**

Most lactobacilli found in animal intestines are generally non-motile, but there are few exceptions. Our previous work showed that *Lactobacillus agilis* BKN88, which is a highly motile strain originating from a chicken, takes advantage of motility in gut colonization in murine models, and thus motile lactobacilli likely have unique ecological characteristics conferred by motility. However, the ecology and habitat of gut-derived motile lactobacilli are still rarely understood. In addition, the limited availability of motile *Lactobacillus* isolates is one of the major obstacles for further studies. To gain insight into the ecology and habitat of the motile lactobacilli, we established a routinely applicable detection method for motile lactobacilli using PCR and subsequent selective isolation in semi-solid MRS medium for the collection of additional motile lactobacilli from animal feces.

**Results:**

We applied the PCR detection using motile lactobacilli-specific primers, based on the motor switch protein gene (*fliG*) of flagella, to 120 animal feces, followed by selective isolation performed using 45 animal feces. As a result, motile lactobacilli were detected in 44 animal feces. In the selective isolation, 29 isolates of *L. agilis* and 2 isolates of *L. ruminis* were obtained from 8 animal species.

**Conclusions:**

These results indicated that motile lactobacilli are distributed in different animal species. Moreover, phylogenetic analysis of the *L. agilis* isolates suggests co-evolution with the host, and adaptation to a particular environmental niche.

## Background

Some lactobacilli are found in the gastrointestinal tract (GIT) of humans and animals and are considered as the gut symbionts [1]. The lactic acid bacteria are known to have specific ecological niches depending on the species/strains. For example, *Lactobacillus johnsonii* and *Lactobacillus reuteri* inhabit the GIT of various animals, but *Lactobacillus gorillae* and *Lactobacillus equigenerosi* exhibit host specificity [2–5]. Additionally the composition of lactobacilli in the gut may be affected by the diets of host animals. Past studies reported that *Lactobacillus brevis, Lactobacillus casei* and *Lactobacillus plantarum* were detected in omnivorous animals but not in carnivores, while *Lactobacillus ingluviei, Lactobacillus salivarius* and *Lactobacillus vaginalis* are dominant in carnivores, but not in most herbivores and omnivores [6]. This suggests that diet has major impacts on the composition of lactobacilli in animal guts.

Motility is a minor characteristic in the genus *Lactobacillus*, and only the small part of the species belonging to the phylogenetic group of *L. salivarius* possesses motility with an exception of the specific species *Lactobacillus curvatus* [7]. Bacterial motility is mostly mediated by flagella. A flagellum is generally composed of approximately 30 different proteins, which form three substructures: filament, hook, and basal body [8]. In motile lactobacilli, the motility-related genes are usually located in single operon, which appears to be conserved [7].

The natural habitats of the motile lactobacilli are variable, such as sewage, freshwater pond, stinky tofu brine, cocoa, cider, wine, shochu, oak tree, grape must, kabura-zushi, chicken, horse and human [7–17]. Of the motile lactobacilli, *L. agilis* and *L. ruminis* are the only known species originating from animal and human guts [18, 19]. The motility related characteristics of the microbes are likely to be involved in their nutrient acquisition and niche colonization. Actually, our previous study showed that *L. agilis* BKN88, which is a highly motile strain isolated from chicken feces, takes advantage of motility and chemotactic ability in gut colonization in murine models [20]. Thus, the motile lactobacilli might have a unique niche. However, gut-derived motile lactobacilli are not well characterized due to lack of studies on motile *Lactobacillus* isolates as well as the limited number of isolated strains in public culture collections [19, 21]. It is thus important to collect additional bacterial strains of motile lactobacilli. However, the selective isolation method of motile lactobacilli has not been developed yet.

In the present study, motile lactobacilli were detected by PCR using motile lactobacilli-specific primers as a culture-independent technique, and then the selective isolation of motile lactobacilli using a soft-agar medium was performed for clarification and confirmation. In addition, we described the general phylogenetic/genomic features of motile *Lactobacillus* isolates.

## Results

### Construction and validation of motile lactobacilli-specific primers

**Fig. 1:**
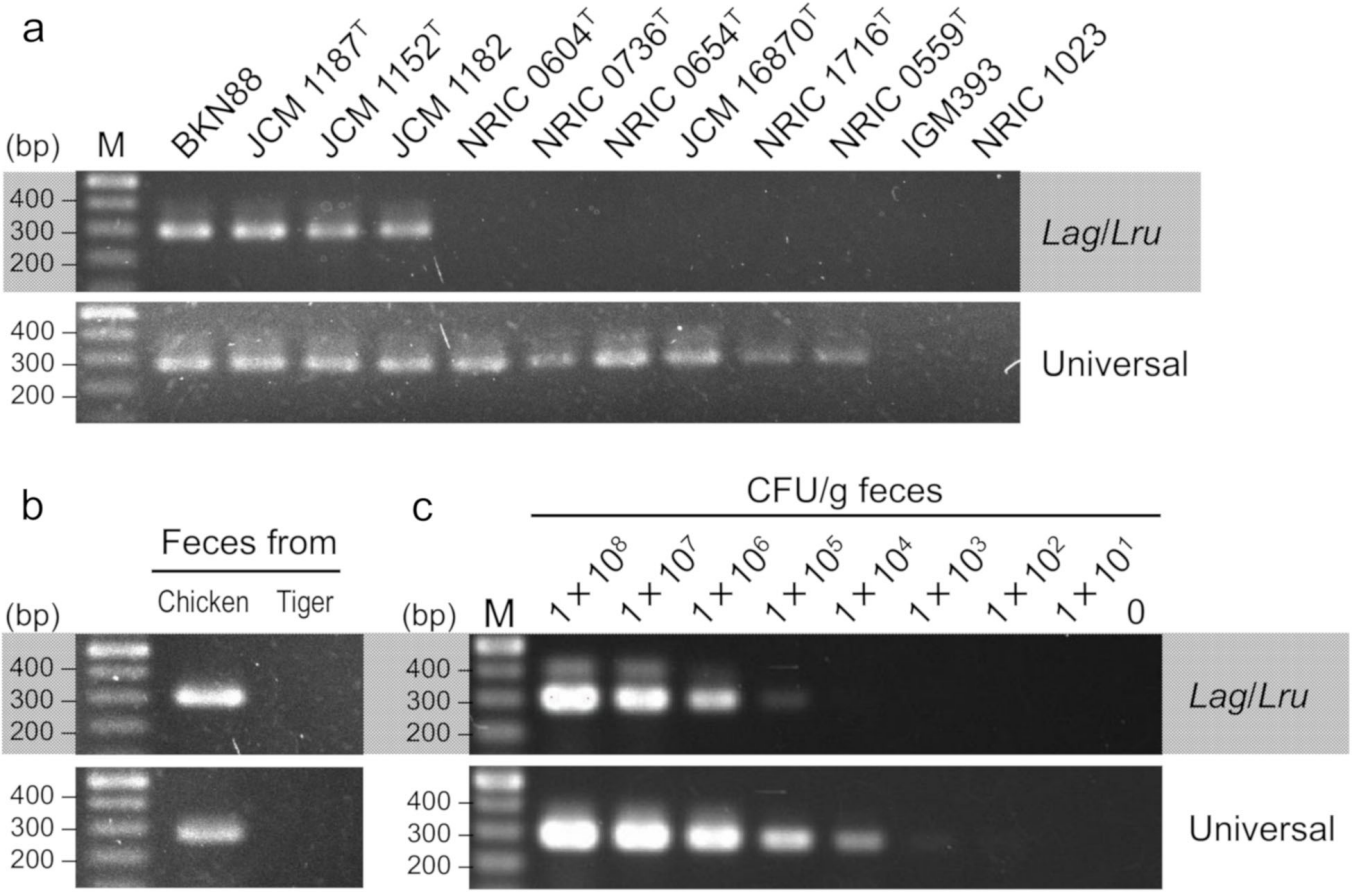
Specificity and sensitivity of PCR detection of motile lactobacilli with *Lag*/*Lru* primers (top) and universal primers (bottom). (**a**) PCR amplification with genomic DNA from eleven strains of motile lactobacilli and a non-motile *Lactobacillus* and an *E. coli*. The strains are listed in Table 2. (**b**) PCR amplification with DNA extracted from feces of a chicken (motile lactobacilli-positive) or a Siberian tiger (motile lactobacilli-negative). (**c**) PCR amplification with DNA extracted from murine fecal pellets supplemented with different concentrations of *L. agilis* BKN88 cells (1 × 10^1^ to 10^8^ CFU/g feces)

Two PCR primer pairs, the first specific to only *L. agilis* and *L. ruminis* (named *Lag*/*Lru* primers) and the second specific to all motile-lactobacilli (named universal primers), targeting the *fliG* gene were designed to detect motile lactobacilli from animal fecal samples (Table 1). To validate the specificity of each primer pair, PCR amplification was performed with genomic DNA from eleven strains of motile lactobacilli including two strains of *L. agilis* and two strains of *L. ruminis*, and a non-motile *Lactobacillus* and *E. coli* (Table 2). In a PCR amplification with the *Lag*/*Lru* primers, *fliG-*specific PCR products were detected from the two strains of *L. agilis* and two strains of *L. ruminis* but not in the non-motile control strains. By using the *fliG* universal primers, PCR products were observed in all motile lactobacilli but not in a non-motile *Lactobacillus* and *E. coli* (Fig. 1a). Furthermore, we tested the primer pairs against chicken and Siberian tiger feces. DNA extracted from chicken feces from which motile lactobacilli could be isolated produced PCR amplicons with each primer pair, but amplicons were not obtained from feces of a Siberian tiger from which motile lactobacilli could not be isolated (Fig. 1b). The sensitivity of each primer pair was tested by PCR amplification with DNA extracted from murine fecal pellets supplemented with serial dilutions of *L. agilis* BKN88 bacterial cells. Since we could not find any motile lactobacilli in the feces from Balb/c mice, the murine fecal pellets were used as motile lactobacilli-free feces [20]. The detection limits of the *Lag*/*Lru* primers and the universal primers were 1 × 10^5^ CFU/g feces and 1 × 10^4^ CFU/g feces, respectively (Fig. 1c).

**Table 1:** Motile lactobacilli-specific primers

Primer	Sequence (5′ to 3′)	Amplicon size (bp)	Annealing temp (°C)
***Lag*/*Lru*primer**		302	60
DOKJ 292	ATCAAGGCGATAGTTTGCGG		
DOKJ 293	TGATTCAGAAGGTCAGTTACG		
**Universal primer**		283	50
DOKJ 294	ACTGTTTGTGGATGTTCATC		
DOKJ 295	AAAGTCAGTTATGAAATTGC

**Table 2:** Bacterial strains used in the validation of primer specificity and sensitivity

Strain or plasmid	Description and Origin	Reference
Strain		
*L. agilis* BKN88	Subculture of JCM 1048, Chicken isolate	[18]
*L. agilis* JCM 1187^T^	Municipal sewage	RIKEN
*L. ruminis* JCM 1152^T^	Mammalian faeces, Bovine rumen	RIKEN
*L. ruminis* JCM 1182	Bovine rumen	RIKEN
*L. satsumensis* NRIC 0604^T^	Mashes of shochu	NRIC
*L. sucicola* NRIC 0736^T^	Sap of oak tree	NRIC
*L. vini* NRIC 0654^T^	Fermenting grape musts	NRIC
*L. uvarum* JCM 16870^T^	Grape musts	RIKEN
*L. curvatus* NRIC 1716^T^	Milk	NRIC
*L. nagelii* NRIC 0559^T^	Partly fermented grape juice	NRIC
*L. paracasei* IGM393	Subculture of ATCC 393, Laboratory strain	[22]
*E. coli* NRIC 1023	Motile strain	NRIC

### PCR detection of motile lactobacilli in animal feces

One hundred twenty fecal samples from various animals were used for screening of motile lactobacilli. As shown in Table 3, the *Lag*/*Lru* primers amplified *fliG* genes from 36 fecal samples, and the universal primers amplified *fliG* genes from 24 fecal samples. By the use of the two primer pairs, *fliG-*specific PCR products were detected in 44 of the 120 fecal samples, which included 25 of the 61 omnivores (41%), 16 of the 52 herbivores (31%) and 3 of the 7 carnivores (43%). In terms of animal species, *fliG-*specific PCR products were detected in 37 of the 93 animal species, which included 20 of the 45 omnivores (44%), 14 of the 41 herbivores (34%) and 3 of the 7 carnivores (43%).

**Table 3:**
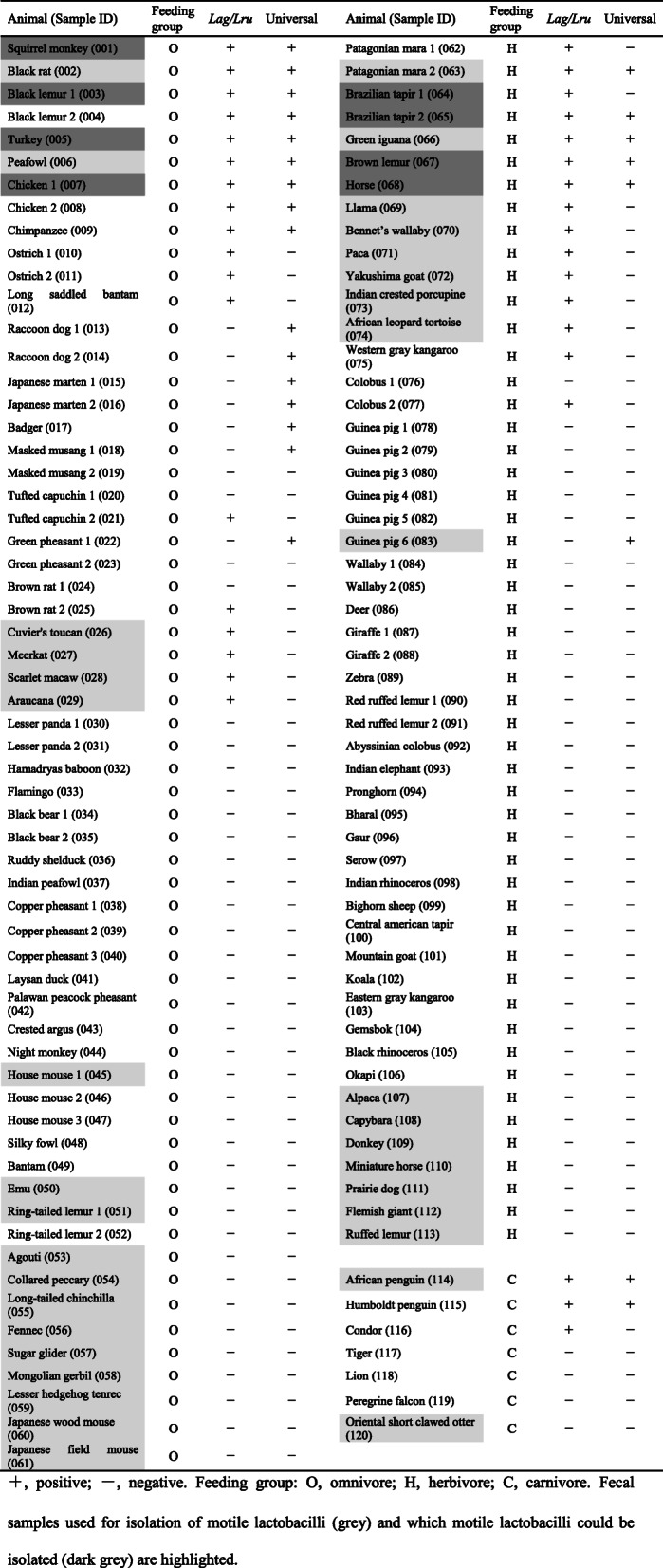
PCR detection of motile lactobacilli in animal feces

+, positive; −, negative

Fecal samples used for isolation of motile lactobacilli (grey) and which motile lactobacilli could be isolated (dark grey) are highlighted

Feeding group: *O* omnivore, *H* herbivore, *C* carnivore

### Isolation of motile lactobacilli from animal feces

The selective isolation of motile lactobacilli using semi-solid MRS medium was applied to only fresh 45 animal feces samples including 23 omnivores, 20 herbivores and 2 carnivores. As shown in Table 4, 29 isolates of *L. agilis* and 2 isolates of *L. ruminis* were obtained from 8 animal species including 5 omnivores and 3 herbivores. No motile lactobacilli were isolated from fecal samples which were negative for PCR detection of *fliG*. In some fecal samples, the motile bacteria were not isolated although *fliG*-specific PCR products were detected. Cloning and sequencing analysis of the amplified *fliG* gene from feces of a Patagonian mara (Sample ID: 063) and Bennett’s wallaby (Sample ID: 070), from which motile bacteria could not be isolated but were positive for *fliG* by PCR, showed that the sequences of the detected *fliG* gene had a 99% sequence similarity with the *fliG* gene of *L. agilis* (GenBank, Accession number KM886859).

**Table 4:** Motile lactobacilli isolated from animal feces

Animal (Sample ID)	Species	Strain	Animal (Sample ID)	Species	Strain
Horse (068)	*L. agilis*	NB11	Brown lemur (067)	*L. agilis*	SN811
	*L. agilis*	NB13	Chicken 1 (007)	*L. agilis*	SN4111
	*L. agilis*	NB14		*L. agilis*	SN4121
	*L. agilis*	NB15		*L. agilis*	SN4211
	*L. agilis*	NB16	Brazilian tapir 1 (064)	*L. agilis*	SY212
	*L. agilis*	NB17		*L. agilis*	SY213
	*L. agilis*	NB18		*L. agilis*	SY2141
	*L. agilis*	NB19		*L. agilis*	SY2142
	*L. agilis*	NB110		*L. agilis*	SY215
	*L. agilis*	NB111	Brazilian tapir 2 (065)	*L. agilis*	KZ171
	*L. agilis*	NB112		*L. agilis*	KZ172
	*L. ruminis*	NB114	Squirrel monkey (001)	*L. agilis*	SN10121
	*L. ruminis*	NB115		*L. agilis*	SN10122
Turkey (005)	*L. agilis*	SY111		*L. agilis*	SN10311
	*L. agilis*	SY121		*L. agilis*	SN10312
Black lemur (003)	*L. agilis*	SN611			

### Phylogenetic analysis based on *flgD* gene sequences

**Fig. 2:**
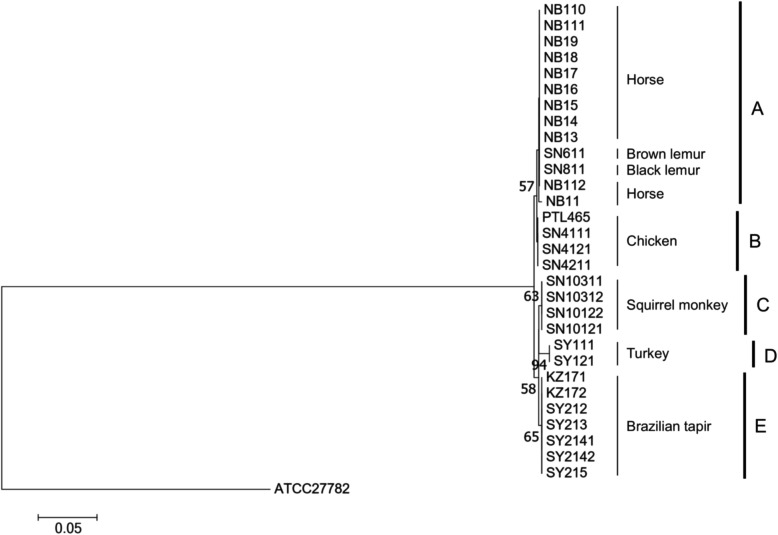
Phylogenetic tree of *L. agilis* isolates based on *flgD* gene sequence. The tree was reconstructed by the neighbour-joining method. *Lactobacillus ruminis* ATCC 27782 was used as an outgroup. Bootstrap percentages above 50% (based on 1000 replications) are shown at branching points

Multiple sequence alignment showed that the *flgD* gene sequence similarities between all pairs of *L. agilis* isolates were at least 98.5%. Subsequent phylogenetic analysis using the neighbor-joining method revealed that *L. agilis* isolates could be differentiated into five groups, designated A-E (Fig. 2). Isolates from the same animal species clustered in the same groups. Only the group of A included the isolates from some animal species, and the isolates from the horse, brown lemur and black lemur shared the group. In the isolates from the brazilian tapirs, KZ strains and SY strains, were obtained from animals in different zoos, however, both isolates belonged to the same group. Chicken isolates in our study were bred in different locations, but were located in the same group as *L. agilis* PTL465. Thus, the representative strains, *L. agilis* NB11, SN4111, SN811, SN10121, SY111 and SY212, from each animal species were used for further analysis.

### Genomic features of *L. agilis* isolates

To obtain more information about the genomic characteristics of *L. agilis* isolates from different animal species, draft genome sequences of seven strains were determined by the Illumina Genome Analyzer II system. Genome sequences of an additional two strains of *L. agilis*, DSM 20509^T^ and La3, were obtained from the NCBI databases for use as references. The GenBank accession numbers of *L. agilis*, DSM 20509^T^ and La3 are AYYP00000000 and CP016766, respectively. The genomic features of all strains used in the present study are summarized in Table 5. All genomes possessed a low contamination level (≤ 2% contamination value), and the completeness values for the all strains of *L. agilis* were 98.23% (Table 5), meaning that all genomes satisfied the criteria to be considered as near-complete genomes with low contamination (≥ 90% completeness value and ≤ 5% contamination value) [23]. The genome size of the type strain (DSM 20509^T^) is 2.06 Mbp and the mol% G + C content of DNA is 41.7 [24]. The genome sizes of the 9 strains of *L. agilis* ranged from 2.03 to 2.52 Mbp and the number of CDSs ranged from 1963 to 2427. The GC contents of the 9 strains of *L. agilis* were 40.5 to 41.7%. *L. agilis* SY212 (Brazilian tapir isolate) had the largest genome and number of CDSs and the lowest GC contents. In a preliminary analysis, we found that the genome of *L. agilis* SY212 contained more putative mobile genetic elements (MGEs) than the other strains of *L. agilis*. These MGEs might be related to the unique genomic features of *L. agilis* SY212. The average nucleotide identity (ANI) values for the tested strains are shown in Additional file 1. ANI values among the 9 strains of *L. agilis* ranged from 0.973 to 0.982.

**Table 5:** General genome characteristics of the strains analyzed

Strains	Source	Size (Mbp)	No. of CDS	No. of Contig	GC (%)	Completeness	Contamination
*L. agilis*							
PTL465	Chicken	2.21	2063	80	41.5	98.23	0.48
NB11	Horse	2.03	1963	75	41.7	98.23	1.29
SN4111	Chicken	2.11	1972	194	41.7	98.23	1.24
SN811	Brown lemur	2.25	2233	81	41.4	98.23	0.86
SN10121	Squirrel monkey	2.14	1993	88	41.7	98.23	1.45
SY111	Turkey	2.26	2129	140	41.4	98.23	0.81
SY212	Brazilian tapir	2.52	2427	239	40.5	98.23	1.75
La3	Chicken	2.19	2114	1	41.6	98.23	1.29
DSM 20509^T^	Municipal sewage	2.05	2014	58	41.7	98.23	1.13

### Carbohydrate utilization of *L. agilis* isolates

Seven strains of *L. agilis* obtained from different animal species were further characterized by sugar fermentation assays using the API 50 CH kit. The carbohydrate fermentation patterns of seven strains are shown in Additional file 2. Different patterns of the acid production from D-mannose, *α*-methyl-D-glucoside, amygdalin, salicin, cellobiose, lactose, trehalose, melezitose, *β*-gentiobiose and D-turanose were shown among the 7 strains of *L. agilis*, but have no obvious correlation to their origins or dietary needs. All *L. agilis* isolates were able to metabolize D-galactose, D-glucose, D-fructose, D-mannitol, *N*-acetylglucosamine, arbutin, esculin, D-maltose, D-lactose, D-melibiose, D-sucrose and D-raffinose.

## Discussion

Some lactobacilli reside in the gut of humans and animals as commensal bacteria [1]. Most of the lactobacilli are non-motile, but a few members of the lactobacilli such as *L. agilis* and *L. ruminis* are motile. Our previous work showed that *L. agilis* takes advantage of motility to survive and colonize in the murine gut [20]. This result implies that motile lactobacilli have unique ecological niches utilizing their motility and chemotactic ability. However, their ecological niches remain unknown. In the present study, we focused on the ecology and habitat of motile lactobacilli.

At least 15 *Lactobacillus* species have been reported as motile lactobacilli to date [7]. Most motile lactobacilli originated from alcoholic fermentation samples, e.g. wine and shochu, whereas *L. agilis* and *L. ruminis* were found in animal and human feces [9, 12, 18]. Thus, two kinds of motile lactobacilli-specific primer pairs were designed to detect fecal-motile lactobacilli and all motile lactobacilli. The motility operon of motile lactobacilli appears to be relatively conserved [7, 21], and the multiple alignment analysis of the motility operons from fourteen publicly available motile lactobacilli showed that the *fliG*, *fliI*, *flhA*, *cheW*, *cheR* and *fliY* genes are highly conserved among the motility operons in the motile lactobacilli. In this study, the most conserved *fliG* gene was used as the target gene for detecting potentially motile lactobacilli. The results of PCR using the designed primer pairs against DNA from various samples demonstrated that these primer pairs may be useful to detect motile lactobacilli.

PCR detection using the currently designed primer pairs were applied to 120 animal feces samples for screening of motile lactobacilli. Consequently, PCR using the *Lag*/*Lru* primers and the universal primers detected motile lactobacilli in 36 samples and 24 samples, respectively.

By the use of the two primer pairs, motile lactobacilli were detected in 44 animal feces samples (37 animal species) including those from animals which have never been reported as origins of motile lactobacilli, and indicated that motile lactobacilli are distributed in various animals. As shown in Table 3, there were differences in detection between the two primer pairs, and the detection of motile lactobacilli could be improved by the use of the two primer pairs. Although the sensitivity of PCR detection of *L. agilis* cells in murine feces using the universal primers was shown to be higher than that of the *Lag*/*Lru* primers, PCR with the *Lag*/*Lru* primers produced amplicons in more animal feces samples than PCR with the universal primers. The reason for this inconsistency might be due to strain-specific nucleotide substitutions at the annealing regions of the universal primers. Further study is essential to improve the inconsistent detection.

In selective isolation of motile lactobacilli using semi-solid MRS medium, 29 isolates of *L. agilis* and 2 isolates of *L. ruminis* were obtained from 8 animal species. Previously, *L. agilis* have been isolated from pigeon, chicken, human, horse, laying hen, piglet and fermented food products such as masau fruits, Nigerian ogi, and cheese [25–34]. *L. ruminis* have been isolated from human, cow, horse and pig [35–41]. Black lemur, brown lemur, turkey, squirrel monkey and brazilian tapir were newly identified as a host of *L. agilis* in this study. All isolates were obtained only from samples positive by PCR for *fliG.* This indicated that the PCR detection using designed primer pairs is a useful/powerful tool to exclude fecal samples negative for motile lactobacilli prior to isolation. In some fecal samples, the motile bacteria were not isolated despite *fliG* specific PCR products having been detected. By sequence analysis, such PCR products were likely to be identified as *L. agilis*. This discrepancy may be because *L. agilis* cells were dead/unculturable or the *L. agilis* strains were not motile at least in the semi-solid MRS medium. In fact, some strains of *L. agilis* and *L. ruminis* do not show motility in MRS medium [38, 42, 43].

In the phylogenetic relationships based on motility-related genes among the 30 *L. agilis* isolates originating from various animal hosts, *L. agilis* isolated from the same host species tended to be clustered together, which suggests the co-evolution with each host. Similar phylogenetic relationships have been observed in other species, such as *L. casei, L. reuteri*, *L. rhamnosus*, *L. johnsoni* and *L. ruminis* [38, 44–47]. Draft genome sequences of seven *L. agilis* isolates were then obtained and analyzed. The ANI values are higher than 95% among the seven isolates, which indicate that the isolated strains may belong to *L. agilis* species. The carbohydrate fermentation patterns of the seven isolates also showed that these strains are highly similar to the type strain of *Lactobacillus agilis* (https://bacdive.dsmz.de/strain/6407). These *L. agilis* isolates and the genome sequences would provide further insight into the ecology of gut-derived motile lactobacilli.

## Conclusions

A combination of PCR detection using *fliG*-specific primer pairs and subsequent isolation with semi-solid MRS medium could successfully isolate a large number of motile lactobacilli from various animal feces, and indicates that motile lactobacilli are distributed in various animals. Phylogenetic analysis on the motility-related gene of *L. agilis* isolates suggests co-evolution with each host, and the adaptation to a particular environmental niche. Additional genomic studies with the obtained sequences need to be done to provide further insights on the ecological adaption of motile lactobacilli in animals and humans.

## Methods

### Bacterial strains and growth conditions

The bacterial strains, listed in Table 2, were obtained from the NODAI Research Institute Culture Collection (NRIC, Tokyo, Japan), RIKEN BioResource Center (JCM, Ibaraki, Japan) and DSMZ German Collection of Microorganisms and Cell Cultures (DSM, Braunschweig, Germany). Thirty-one motile lactobacilli isolated from animal feces are described in Table 4. All *Lactobacillus* strains were propagated anaerobically in MRS broth (Difco/BD) at 37 °C. Motilities of *Lactobacillus* strains were determined by visual examination after inoculation into semi-solid MRS medium with 0.15% agar. *E. coli* NRIC 1023 and *E. coli* JM109 were grown aerobically in LB medium with or without 100 μg/ml of ampicillin and 25 μg/ml of kanamycin at 37 °C.

### Design of motile lactobacilli-specific primers

In order to design the motile lactobacilli-specific primers, DNA sequences of the motility operons from fourteen motile lactobacilli were obtained from the NCBI database and analyzed by multiple alignments using ClustalW [48]. The highly conserved *fliG* gene, which encodes the flagellar motor switch protein of the motility operon, was targeted for the detection of motile lactobacilli in animal feces. Two PCR primer pairs targeting the *fliG* gene were designed. One primer pair was designed to amplify the *fliG* gene specifically in *L. agilis* and *L. ruminis,* while another was designed to amplify the *fliG* gene of most motile lactobacilli. These primer pairs are referred to as *Lag*/*Lru* primers and universal primers, respectively. The reason we prepared the *Lag*/*Lru* primers is because some strains of *L. agilis* and *L. ruminis* have been previously isolated from animal or human feces [18, 19]. Motile lactobacilli-specific primers are listed in Table 1. The primer pairs *Lag*/*Lru* primers and universal primers produce an approximately 300 bp and 280 bp PCR amplicon, respectively.

### Validation of primer specificity and sensitivity

The specificity of each primer pair was determined by PCR amplification with genomic DNA from ten strains of motile lactobacilli including two strains of *L. agilis* and two strains of *L. ruminis*, *Lactobacillus paracasei* IGM393 and *Escherichia coli* NRIC 1023 as templates. DNA from bacterial cultures was prepared as follows. Bacteria cells from an overnight culture were harvested and washed with TE buffer, followed by bead beating with a FastPrep Instrument (MP Biomedicals) in TE buffer. Then, DNA was extracted using phenol–chloroform and ethanol precipitation according to standard protocols. Further determination of the specificity of each primer pair was performed with PCR amplification using DNA extracted from feces of a chicken or a Siberian tiger. In past studies, motile lactobacilli were found in a chicken but not in a Siberian tiger [32]. In this study we also were able to isolate motile lactobacilli from a chicken but not from a Siberian tiger. Therefore the feces from a chicken and Siberian tiger were used as motile lactobacilli-positive or -negative fecal samples, respectively. To validate the sensitivity of each primer pair, PCR amplification was carried out with DNA extracted from murine fecal pellets supplemented with different concentrations of *L. agilis* BKN88 cells (1 × 10^1^ to 10^8^ CFU/g feces). Murine fecal pellets were collected from Balb/c mice, which do not have motile lactobacilli in their feces. Balb/c mice were obtained from Crea Japan, Inc. DNA extraction and PCR amplification from fecal samples are described below.

### Fecal samples and DNA extraction

Fecal samples were collected from 120 animals from various zoos in Japan. Based on their dietary needs, they were classified as omnivores, herbivores or carnivores, listed in Table 3. The samples were collected in sterile plastic tubes and immediately taken to the laboratory under refrigerated conditions. The fecal samples were stored at − 20 °C until DNA extraction. DNA was extracted from 200 mg of fecal pellet with QIAamp DNA Stool Mini Kit (Qiagen), according to the manufacturer’s instructions.

### PCR amplification

PCR amplification was performed using Ex *Taq* DNA polymerase (Takara). PCR was carried out in a total volume of 25 μl, containing 2.5 μL of 10× Ex Taq buffer, 2 μL of dNTP mixture (2.5 mM each), 2.5 μl of each of primers (10 μM), 0.25 μl of *TaKaRa Ex Taq* (5 U/μl) and 0.5 μl of extracted DNA. PCR conditions were as follow: 5 min at 94 °C; followed by 30 cycles of 30 s at 94 °C, 30 s at 60 or 50 °C, 10 s at 72 °C and a final extension step of 5 min at 72 °C. The PCR with *Lag*/*Lru* primers and universal primers was performed at an annealing temperature of 60 and 50 degrees, respectively. The PCR products were separated in 1.5% agarose gel by electrophoresis. DNA bands were visualized by staining with ethidium bromide.

### Isolation of motile lactobacilli

Fecal samples which were used for isolation of motile lactobacilli are highlighted in Table 3. Fecal samples for isolation of motile lactobacilli were suspended in sterile PBS, and homogenized using a vortex mixer. The fecal suspensions were inoculated into semi-solid MRS medium with 0.15% agar, and incubated at 37 °C for 1 day. The migrated cells from the outermost ring were collected and inoculated onto MRS agar plates, and the plates were incubated under anaerobic conditions at 37 °C for 1 to 2 days. Isolated colonies were randomly selected from the surface of MRS agar plates and restreaked onto MRS agar plates, and incubated under anaerobic condition at 37 °C for 1 day as before. Isolates were purified by several streakings. Identification of the bacterial isolates was carried out by Sanger sequencing of the 16S rRNA gene. PCR amplification with 16S primers [49], 27F (5′- AGA GTT TGA TCC TGG C-3′) and 1492R (5′- CGG TTA CCT TGT TAC G-3′), was performed with Ex *Taq* DNA polymerase (Takara). The PCR products were purified using the NucleoSpin Gel and PCR Clean-up kit (Macherey-Nagel) according to the manufacturer’s instructions and sequenced by Macrogen Japan (Tokyo, Japan) with the primers 27F and 1492R. The 16S rRNA gene was sequenced for each isolate and used for BLAST analysis using GenBank.

### Cloning and DNA sequencing of the *fliG* gene

The *fliG* genes were amplified using Ex *Taq* DNA polymerase (Takara) and the *Lag*/*Lru* primers with DNA extracted from feces of a Patagonian mara (Sample ID: 063) and Bennett’s wallaby (Sample ID: 070), from which the motile bacteria were not isolated, although *fliG* specific PCR products were detected. Purification of PCR products from 1.5% agarose gels was performed with the NucleoSpin Gel and PCR Clean-up kits (Macherey-Nagel) according to the manufacturer’s instructions. These PCR products were inserted into the pGEM-T Easy Vector (Promega) using T4 DNA Ligase (Takara). *E. coli* JM109 was used as a cloning host. The constructed plasmid was purified with the NucleoSpin Plasmid EasyPure kit (Macherey-Nagel). The sequences of the insert DNA were determined by Sanger sequencing with the M13 primers M13F (5′-GTA AAA CGA CGG CCA GT-3′) and M13R (5′-CAG GAA ACA GCT ATG AC-3′). The result of the sequencing was analyzed as described above.

### Sequencing of *flgD* gene and phylogenetic analysis

The motility operons from four strains of *L. agilis* publicly available in databases were analyzed by multiple alignments using ClustalW. The highly conserved *flgD, motA, flgE, flgG* and *fliM* genes were selected for the phylogenetic analysis of fecal isolates; however, the *motA, flgE, flgG* and *fliM* genes could not be amplified in some of the isolates. Thus, the *flgD* genes were used for further analysis. The *flgD* genes were amplified using primers DOKJ507 (5′-AAT TTA AGT GAT GCG GTA GC-3′) and DOKJ508 (5′-ATT TGG CAT CGC CTA CTT GG-3′) with DNA extracted from *L. agilis* isolates. The sequences of the *flgD* genes were determined with DOKJ507 and DOKJ508 as explained above. Approximately 355 bp of *flgD* gene sequences of the isolates and related strains or species were used in the phylogenetic analysis. The *flgD* gene of *L. ruminis* ATCC 27782 was used as an outgroup. Multiple sequence alignment was carried out with ClustalW [48], and the phylogenetic tree was generated by the neighbor-joining method with 1000 bootstrap replications using MEGA7 [50].

### Draft genome sequencing and denovo assembly

Genomic DNA from seven strains of *L. agilis*, PTL465, NB11, SN4111, SN811, SN10121, SY111 and SY212, was isolated using the DNAiso Reagent (Takara) according to the manufacturer’s instructions. Whole-genome sequencing was performed using Illumina MiSeq, with an insert length of approximately 500 bp. A total of 1,045,376, 1,101,945, 1,151,675, 874,420, 929,125, 1,064,309, 1,007,553 reads with average length of 151 bps was obtained from *L. agilis*, PTL465, NB11, SN4111, SN811, SN10121, SY111 and SY212, respectively. Draft genomes were assembled using Platanus_B (version 1.1.0) [51] with default settings. Sequences shorter than 300 bp were eliminated. The genome was annotated using the DDBJ Fast Annotation and Submission Tool (DFAST, https://dfast.nig.ac.jp) [52]. The completeness and contamination of the genomic data were assessed by CheckM (Version 1.0.4) [23]. Average nucleotide identity (ANI) was calculated as the mean identity of pair-wise sequence alignment between two genomes [53].

### Fermentation profiles of *L. agilis* isolates

To determine carbohydrate utilization profiles, API 50 CH (bioMérieux) tests were used according to the manufacturer’s instructions. Data for the reference type strain (DSM 20509^T^) was taken from the BacDive database at https://bacdive.dsmz.de/strain/6407.

### Accession numbers

The draft genome sequences of *L. agilis* SY212, SY111, SN10121, SN811, SN4111, NB11 and PTL465 were deposited in DDBJ/EMBL/GenBank under the accession numbers BLAM01000001-BLAM01000239, BLAN01000001-BLAN01000140, BLAO01000001-BLAO01000088, BLAP01000001-BLAP01000081, BLAQ01000001-BLAQ01000194, BLAR01000001-BLAR01000075 and BLAS01000001-BLAS01000080.

## Supplementary information

**Additional file 1.** ANI values obtained for the tested strains.

**Additional file 2 **Carbohydrate utilization of *L. agilis* isolates.

## Data Availability

The datasets during and/or analysed during the current study available from the corresponding author on reasonable request.

## Abbreviations

PCR: Polymerase chain reaction
GIT: Gastrointestinal tract
MRS: Man, Rogosa and Sharpe
LB: Luria-Bertani
CFU: Colony forming units
16S rRNA: 16S ribosomal RNA
ANI: Average nucleotide identity
CDS: Coding sequence
