# Supplementary material for: PCR-based screening, isolation, and partial characterization of motile lactobacilli from various animal feces
Source: BMC Microbiol. 2020 Jun 3;20:142. doi: 10.1186/s12866-020-01830-7 (PMC7268542; doi:10.1186/s12866-020-01830-7)
Supplement: Supplementary file 1 — Additional file 1. ANI values obtained for the tested strains. [file 12866_2020_1830_MOESM1_ESM.docx]

| query | DSM20509 | La3 | PTL465 | NB11 | SN4111 | SN811 | SN10121 | SY111 | SY212 |
| --- | --- | --- | --- | --- | --- | --- | --- | --- | --- |
| DSM20509 |  | 0.9755 | 0.9794 | 0.9783 | 0.9757 | 0.9793 | 0.9787 | 0.9761 | 0.9770 |
| La3 | 0.9746 |  | 0.9761 | 0.9752 | 0.9773 | 0.9747 | 0.9757 | 0.9746 | 0.9758 |
| PTL465 | 0.9780 | 0.9754 |  | 0.9768 | 0.9759 | 0.9787 | 0.9795 | 0.9758 | 0.9784 |
| NB11 | 0.9779 | 0.9758 | 0.9778 |  | 0.9747 | 0.9818 | 0.9781 | 0.9752 | 0.9770 |
| SN4111 | 0.9752 | 0.9763 | 0.9754 | 0.9730 |  | 0.9745 | 0.9741 | 0.9727 | 0.9745 |
| SN811 | 0.9785 | 0.9745 | 0.9791 | 0.9815 | 0.9748 |  | 0.9789 | 0.9748 | 0.9773 |
| SN10121 | 0.9778 | 0.9750 | 0.9785 | 0.9768 | 0.9741 | 0.9782 |  | 0.9767 | 0.9789 |
| SY111 | 0.9750 | 0.9742 | 0.9761 | 0.9743 | 0.9729 | 0.9758 | 0.9774 |  | 0.9754 |
| SY212 | 0.9756 | 0.9745 | 0.9778 | 0.9762 | 0.9737 | 0.9766 | 0.9784 | 0.9748 |  |

Table S1. ANI values obtained for the tested strains
