# Supplementary material for: PCR-based screening, isolation, and partial characterization of motile lactobacilli from various animal feces
Source: BMC Microbiol. 2020 Jun 3;20:142. doi: 10.1186/s12866-020-01830-7 (PMC7268542; doi:10.1186/s12866-020-01830-7)
Supplement: Supplementary file 2 — Additional file 2 Carbohydrate utilization of L. agilis isolates. [file 12866_2020_1830_MOESM2_ESM.docx]

Table S2. Carbohydrate utilization of *L. agilis* isolates

| Carbohydrates | PTL465 | NB11 | SN4111 | SN811 | SN10121 | SY111 | SY212 |
| --- | --- | --- | --- | --- | --- | --- | --- |
| Glycerol | － | － | － | － | － | － | － |
| Erythritol | － | － | － | － | － | － | － |
| D-Arabinose | － | － | － | － | － | － | － |
| L-Arabinose | － | － | － | － | － | － | － |
| D-Ribose | w | w | w | w | w | w | w |
| D-Xylose | － | － | － | － | － | － | － |
| L-Xylose | － | － | － | － | － | － | － |
| D-Adonitol | － | － | － | － | － | － | － |
| Methyl-*β*  D-xylopyranoside | － | － | － | － | － | － | － |
| D-Galactose | ＋ | ＋ | ＋ | ＋ | ＋ | ＋ | ＋ |
| D-Glucose | ＋ | ＋ | ＋ | ＋ | ＋ | ＋ | ＋ |
| D-Fructose | ＋ | ＋ | ＋ | ＋ | ＋ | ＋ | ＋ |
| D-Mannose | ＋ | ＋ | ＋ | ＋ | ＋ | ＋ | w |
| L-Sorbose | － | － | － | － | － | － | － |
| L-Rhamnose | － | － | － | － | － | － | － |
| Dulcitol | － | － | － | － | － | － | － |
| Inositol | － | － | － | － | － | － | － |
| D-Mannitol | ＋ | ＋ | ＋ | ＋ | ＋ | ＋ | ＋ |
| D-Sorbitol | － | － | － | － | － | － | － |
| Methyl-*α*  D-mannopyranoside | － | － | － | － | － | － | － |
| Methyl-*α*  D-glucopyranoside | － | － | － | － | ＋ | － | － |
| *N*-Acetylglucosamine | ＋ | ＋ | ＋ | ＋ | ＋ | ＋ | ＋ |
| Amygdalin | w | ＋ | w | ＋ | w | w | － |
| Arbutin | ＋ | ＋ | ＋ | ＋ | ＋ | ＋ | ＋ |
| Esculin | ＋ | ＋ | ＋ | ＋ | ＋ | ＋ | ＋ |
| Salicin | ＋ | ＋ | ＋ | ＋ | ＋ | ＋ | w |
| D-Cellobiose | ＋ | ＋ | w | ＋ | ＋ | ＋ | ＋ |
| D-Maltose | ＋ | ＋ | ＋ | ＋ | ＋ | ＋ | ＋ |
| D-Lactose | w | ＋ | ＋ | ＋ | ＋ | ＋ | ＋ |
| D-Melibiose | ＋ | ＋ | ＋ | ＋ | ＋ | ＋ | ＋ |
| D-Sucrose | ＋ | ＋ | ＋ | ＋ | ＋ | ＋ | ＋ |
| D-Trehalose | ＋ | ＋ | ＋ | ＋ | ＋ | ＋ | － |
| Inulin | － | － | － | － | － | － | － |
| D-Melezitose | － | － | － | － | ＋ | － | － |
| D-Raffinose | ＋ | ＋ | ＋ | ＋ | ＋ | ＋ | ＋ |
| Starch | － | － | － | － | － | － | － |
| Glycogen | － | － | － | － | － | － | － |
| Xylitol | － | － | － | － | － | － | － |
| Gentiobiose | ＋ | ＋ | － | ＋ | － | ＋ | － |
| D-Turanose | － | － | － | w | － | － | ＋ |
| D-Lyxose | － | － | － | － | － | － | － |
| D-Tagatose | － | － | － | － | － | － | － |
| D-Fucose | － | － | － | － | － | － | － |
| L-Fucose | － | － | － | － | － | － | － |
| D-Arabitol | － | － | － | － | － | － | － |
| L-Arabitol | － | － | － | － | － | － | － |
| Gluconate | － | － | － | － | － | － | － |
| 2-keto-gluconate | － | － | － | － | － | － | － |
| 5-keto-gluconate | － | － | － | － | － | － | － |

＋, positive; －, negative; w, weakly positive.
